# Hairy Cell Leukemia Presenting with Isolated Skeletal Involvement Successfully Treated by Radiation Therapy and Cladribine: A Case Report and Review of the Literature

**DOI:** 10.1155/2015/803921

**Published:** 2015-12-16

**Authors:** Ipek Yonal-Hindilerden, Fehmi Hindilerden, Sanem Bulut-Dereli, Eren Yıldız, Ibrahim Oner Dogan, Meliha Nalcaci

**Affiliations:** ^1^Istanbul Medical Faculty, Department of Internal Medicine, Division of Hematology, Istanbul University, 34104 Istanbul, Turkey; ^2^Hematology Clinic, Bakırkoy Sadi Konuk Training and Research Hospital, 34147 Istanbul, Turkey; ^3^Department of Radiology, Istanbul Bilim University, 34394 Istanbul, Turkey; ^4^Istanbul Medical Faculty, Department of Orthopaedics and Traumatology, Istanbul University, 34104 Istanbul, Turkey; ^5^Istanbul Medical Faculty, Department of Pathology, Istanbul University, 34104 Istanbul, Turkey

## Abstract

We describe an unusual case of hairy cell leukemia (HCL) in a 55-year-old male presenting with isolated skeletal disease as the initial manifestation without abnormal peripheral blood counts, bone marrow involvement, or splenomegaly. To the best of our knowledge, there have been only two previous reports of a similar case. The patient presented with pain in the right femur. Anteroposterior radiographs of both femurs revealed mixed lytic-sclerotic lesions. PET scan showed multiple metastatic lesions on axial skeleton, pelvis, and both femurs. Histopathological examination of the bone biopsy revealed an infiltrate of HCL. Localized radiation therapy to both proximal femurs and subsequently 4 weeks later, a 7-day course of 0.1 mg/kg/day cladribine provided complete remission with relief of symptoms and resolution of bone lesions. We addressed the manifestations and management of HCL patients with skeletal involvement.

## 1. Introduction

Hairy cell leukemia (HCL) is an uncommon indolent B-cell lymphoproliferative disease that typically affects middle-aged men with a median age of diagnosis of 52 years. HCL is characterized by sensitivity to treatment with either *α*-interferon or purine analog-based therapy such as cladribine. In 1958, HCL was first defined as a distinct clinicopathologic entity by Bouroncle et al. [1]. HCL represents about two percent of all leukemias and is characterized by infiltration of peripheral blood, bone marrow, and spleen by malignant cells with prominent irregular cytoplasmic projections, namely, “hairy cells” [2]. Usually, patients with HCL respond well to purine analog-based therapy. HCL patients may be asymptomatic or develop symptoms of cytopenia, particularly infections [3]. Patients usually present with splenomegaly (80–90%), pancytopenia (60–80%) and hepatomegaly (40–50%). Unusual sites of involvement include abdominal lymph nodes (10%), skin, serosa, meninges, kidney, eye, and pancreas [1, 4–6]. Skeletal involvement is a rare complication of HCL. The incidence of skeletal involvement in HCL is reported as 3% in the literature [7, 8]. Skeletal lesions are commonly lytic and the most common sites of involvement are the femoral head and neck [9]. Lembersky et al. reported that skeletal involvement is typically associated with high tumor burden [8]. While bone marrow infiltration is the rule in HCL, we reported a rare case of HCL in a patient with isolated skeletal disease and no splenomegaly or bone marrow involvement. To the best of our knowledge, there are only several case reports in the literature of a similar case [10, 11].

## 2. Case Presentation

**Figure 1:**
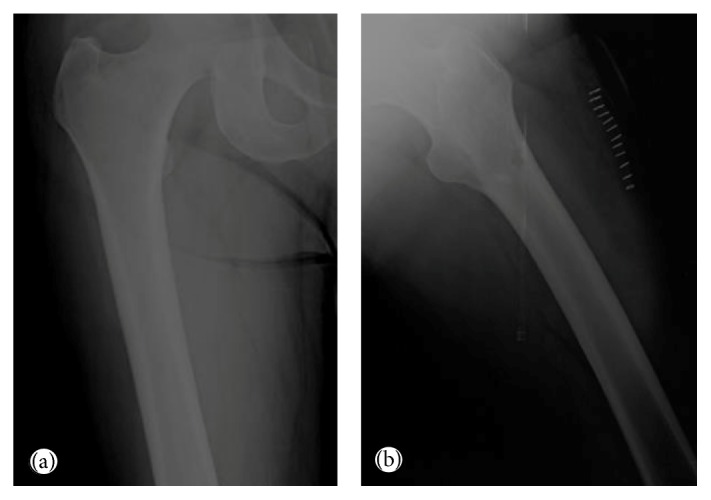
Anteroposterior radiographs of both femurs. (a) Mixed lytic-sclerotic lesions on the right femur. (b) Mixed lytic-sclerotic lesions on the left femur and a lytic destructive process with eccentric localization and a narrow zone of transition at diaphysis of the left femur extending to the left subtrochanteric region.

**Figure 2:**
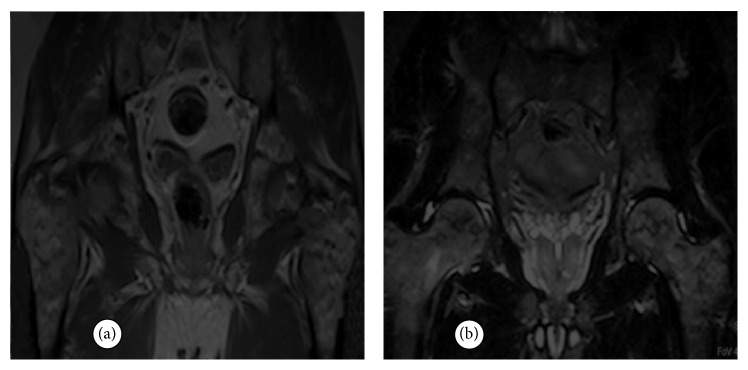
MRI scan of pelvis and both femurs showed multiple metastatic bone lesions dominantly located in both femoral heads, necks, and trochanters with a largest of diameter 2 cm. (a) Multiple hypointense metastatic bone lesions on T1-weighted coronal imaging. (b) Multiple hyperintense metastatic bone lesions on T2-weighted coronal imaging.

**Figure 3:**
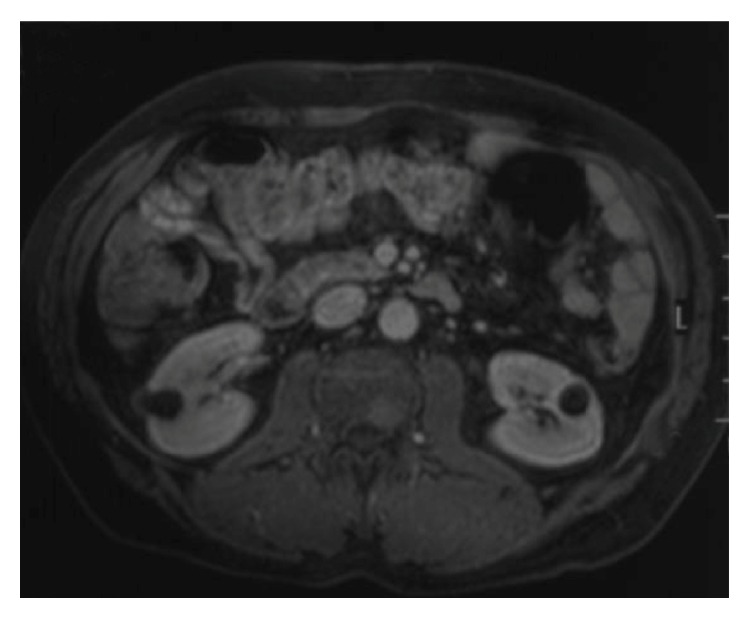
Abdominal MRI on T1-weighted axial imaging revealed a 1 cm wide heterogeneous metastatic lesion at the left side of posterior L2 and L3 vertebrae corpus.

**Figure 4:**
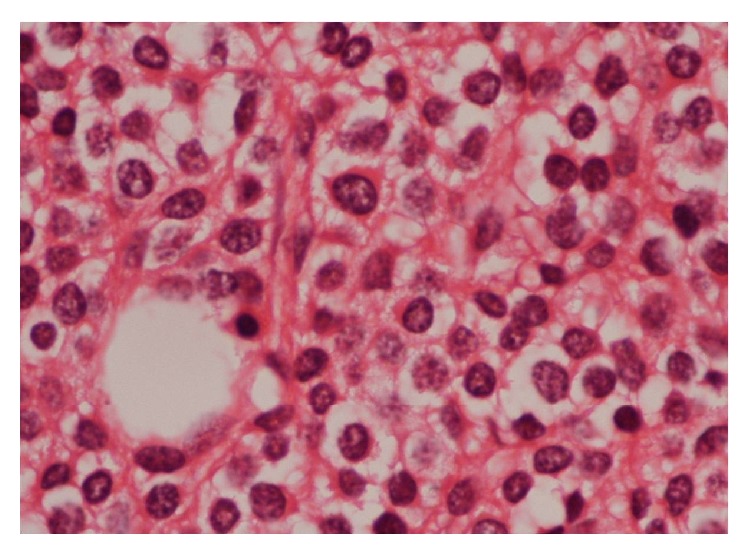
The biopsy specimen of the right femoral lesion showed diffuse neoplastic infiltration consisting of cells with round, oval, regular nuclei, and medium-sized, clear cytoplasm (H&E, ×400).

**Figure 5:**
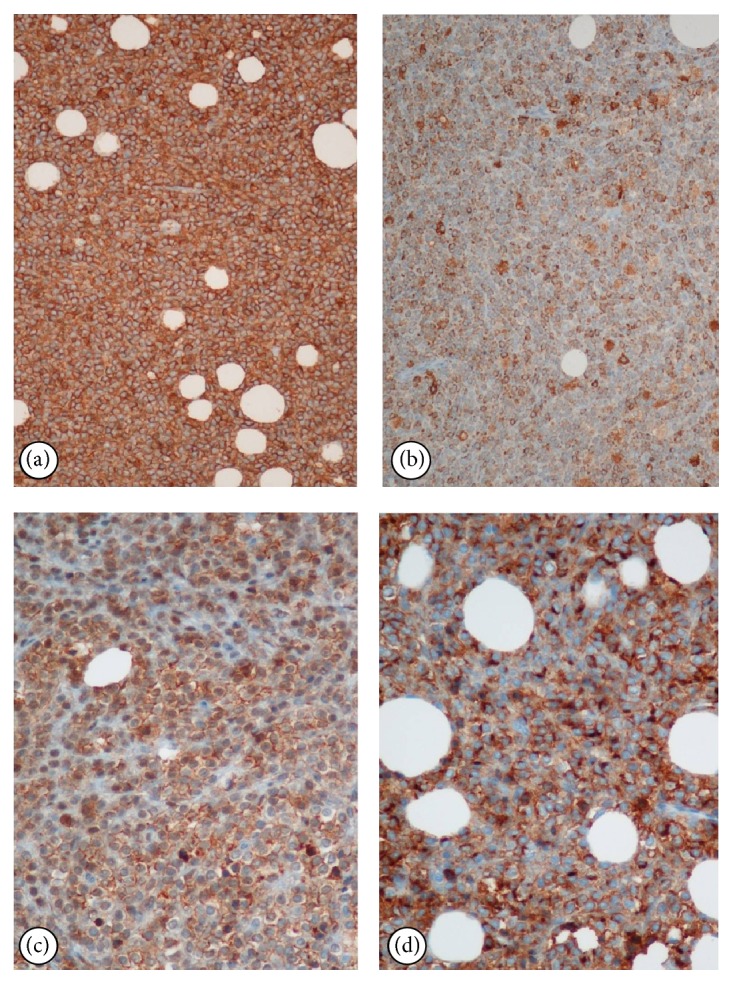
Immunohistochemical features of the infiltrated cells in the right femur expressed CD20 ((a) ×200), TRAP ((b) ×200), CD11c ((c) ×400), and annexin ((d) ×400).

**Figure 6:**
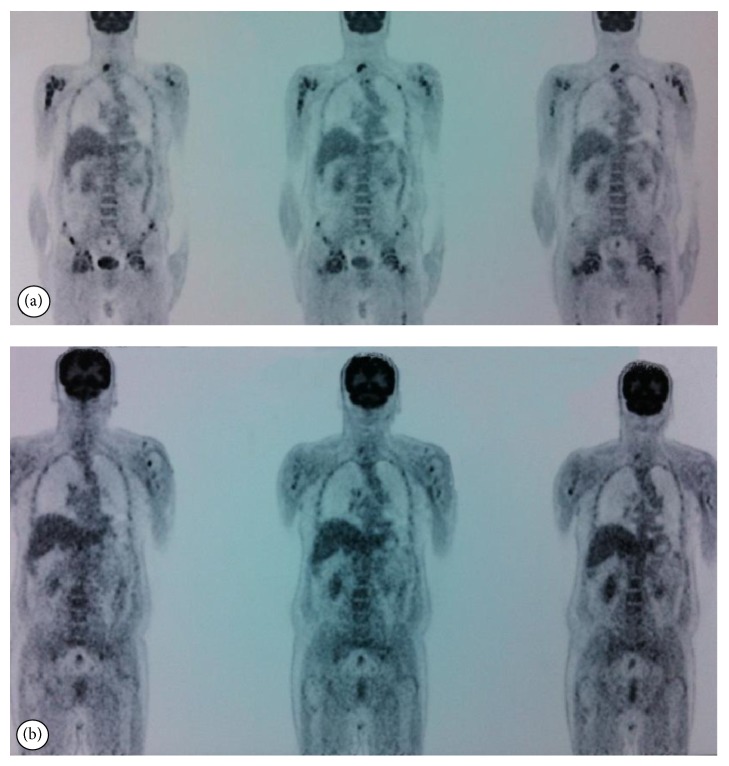
PET scan at diagnosis and 8 weeks after chemotherapy. (a) There was increased FDG uptake on axial skeleton, pelvis, and both femurs at diagnosis. (b) Follow-up PET scan demonstrated marked metabolic response with decrease in uptake of multiple metastatic lesions on both proximal humeri, the right lamina of C6 vertebra, and both first ribs.

A 55-year-old male was admitted to the orthopaedics and traumatology clinic with a 3-month history of severe pain in the right femur extending to the right leg. His medical history was insignificant. On physical examination, the patient's right leg was tender to palpation, particularly in the area of the proximal femur, and there was limited range of motion of the right hip. Blood count was as follows: haemoglobin: 13 g/dL, total leukocyte count: 6030/mm^3^ (neutrophil 58%, lymphocyte 34%), and platelet: 267000/mm^3^. Biochemical tests showed increased erythrocyte sedimentation rate (ESR) of 50 mm/h and hypergammaglobulinemia (gamma globulin 1.56 g/dL); lactate dehydrogenase (LDH) was normal. Anteroposterior radiographs of both femurs revealed mixed lytic-sclerotic lesions (Figures 1(a) and 1(b)) and a lytic destructive process with eccentric localization and a narrow zone of transition at diaphysis of left femur extending to left subtrochanteric region (Figure 1(b)). On MRI scan, multiple hypointense metastatic bone lesions on T1-weighted coronal imaging and hyperintense metastatic lesions on T2-weighted coronal imaging dominantly located in both femoral heads, necks, and trochanters with largest of diameter 2 cm were present (Figures 2(a) and 2(b)). Abdominal MRI on T1-weighted axial imaging revealed a 1 cm wide heterogeneous metastatic lesion at left side of posterior L2 and L3 vertebrae corpus (Figure 3). Histopathological examination of the biopsy from right femoral lesion showed diffuse neoplastic infiltration consisting of cells with round, oval, regular nuclei, and medium-sized, clear cytoplasm (Figure 4). Neoplastic cells expressed CD20, LCA, CD79 alpha, tartrate-resistant acid phosphatase (TRAP), CD11c, CD68, and annexin (Figure 5). BRAF V600E mutation was positive. Diagnosed with HCL, the patient was referred to our hematology department. Blood smear showed 54% neutrophils, 40% lymphocytes, and 6% monocytes. Lymphocytes appeared normal. Bilateral bone marrow aspirates revealed normal bone marrow elements with no hairy cells. Flow cytometry performed on bilateral bone marrow aspirates revealed no abnormal clone of lymphoid cells or aberrant antigen expression. Bilateral bone marrow trephine biopsies showed a normocellular bone marrow with no abnormal infiltrates. On PET scan, there was increased FDG uptake on lateral sides of right 2nd and 3rd ribs, posterior sides of right 9th and 10th ribs, lateral sides of left 2nd, 5th, 6th, and 7th ribs, corpus sterni, corpus of D1, D5, D6, L1, and L2 vertebrae, spinous processes of D12, L3, and L4 vertebrae, right pedicle of C6 and D11 vertebrae, both femoral heads, necks and diaphyseal regions, sacrum, and pelvic bones (Figure 6(a)). Patient underwent radiotherapy to bilateral proximal femur at a dose of 20 Gy and obtained a complete clinical response with resolution of pain. Four weeks after completion of radiotherapy, a 7-day course of 0.1 mg/kg/day cladribine was administered. Follow-up PET scan performed 8 weeks after chemotherapy showed marked metabolic response with decrease in uptake of multiple metastatic lesions on both proximal humeri, right lamina of C6 vertebrae, and both first ribs (Figure 6(b)). Repeat PET scan 12 months after chemotherapy showed complete recovery of skeletal disease with no evidence of residual enhancement suggestive of disease. There is no sign of disease recurrence 14 months after completion of chemotherapy.

## 3. Discussion

HCL is an indolent disorder of B-cell origin that accounts for 2% of all forms of leukemia and 8% of lymphoproliferative diseases [3]. The clinicopathologic entity now named HCL was first described as leukemic reticuloendotheliosis by Bouroncle et al. with a 4.2 : 1 male dominance, with an age range of 33–76 years [1]. In 1966, it was named HCL based on the observation of malignant cells with prominent irregular cytoplasmic projections [12]. Diagnosis is based on the distinctive hairy cell morphology with the presence of large numbers of mononuclear cells with cytoplasmic projections in the blood and/or bone marrow [13]. Bone marrow is hypercellular in most cases and hairy cell infiltration may be diffuse, focal, or interstitial. Hairy cells strongly express pan-B-cell antigens including CD19, CD20, CD22, and CD25 and characteristically express CD11c, CD103, CD123, cyclin D1, and annexin A1 and usually lack expression of CD5, CD10, CD21, and CD23 [14, 15]. BRAF is a commonly mutated gene in a variety of cancers. BRAF V600E is the most common mutation reported in exon 15 of BRAF [16, 17]. Although BRAF V600E mutation is not detected in other B-cell malignancies, until now almost all cases of HCL display BRAF V600E mutation. Therefore, it is thought that BRAF V600E mutation may be considered as a hallmark of HCL [18]. Recently, CDNK1B was reported as the second most common mutated gene in HCL. Somatic CDKN1B (p27) mutations were identified in 16% of HCL patients and coexist with BRAF V600E mutations [19].

Approximately one-quarter of HCL patients are asymptomatic. Some patients can be observed for months or years after the initial diagnosis, which can be made after an incidental finding of splenomegaly or cytopenias during evaluation for an unrelated cause. One-quarter of patients present with abdominal fullness due to splenomegaly [14]. In 14% of the patients, the spleen is moderately enlarged and in 42%, it is massive [20]. Another one-quarter of patients present with systemic complaints such as weight loss, weakness, and fatigue. Fever or night sweats are unusual symptoms. Leukopenia is seen in 58% of patients and marked thrombocytopenia (<100,000/mm^3^) in 46% of patients [20]. One-quarter of patients present either with recurrent infections secondary to leukopenia or with bruising and bleeding secondary to severe thrombocytopenia. Hairy cells typically infiltrate the bone marrow, peripheral blood, spleen, and liver. Most common findings are splenomegaly (60–70%) and hepatomegaly (40–50%). Lymphadenopathy present in about 10 percent of patients is not a major feature of HCL [3]. Other reported uncommon features of disease include skin [21] and neurologic involvements [22], rheumatologic diseases/vasculitis [23], gastric infiltration, ascites [5], nephrotic syndrome [24], scleroderma [25, 26], sarcoidosis [27], and retinal vasculitis/uveitis [28].

Skeletal involvement is an uncommon disease complication. Clinical features of reported cases of HCL with skeletal involvement, including our case, are summarized in Table 1. Quesada et al. reported four patients with osseous involvement among 46 patients with HCL [4]. Manifestations included osteolytic lesions, severe osteoporosis, and aseptic necrosis of the femoral head. Demanes et al. reported two patients, one of whom initially presented with lytic lesions in the right femoral neck and the other is characterized by development of lytic lesions in the right femoral neck, upper and lower thoracic vertebrae, and L2 vertebral body 3 years after initial diagnosis [29]. Lembersky et al. identified eight patients with osseous complications associated with HCL [8]. The lesions were primarily lytic and located in the proximal femur. All patients had bone marrow infiltration with hairy cells. This study indicated that bone involvement is associated with high tumor burden in the bone marrow and suggested that, in such cases in addition to local radiation therapy, systemic treatment with interferon should be considered. In our case, however, there was skeletal involvement without bone marrow infiltration. Herold et al. defined two patients: one with lytic lesions of the right femoral head and neck complicated by a pathologic fracture and the other with osteolytic and osteoblastic lesions developed in multiple thoracic and lumbar vertebral bodies and a compression fracture of the 12th thoracic vertebra [9]. In our case, lesions were mixed lytic-sclerotic and sites of involvement included axial skeleton, sacrum, pelvis, and both femurs. Snell et al. reported a patient who presented with a pathological femoral fracture 22 years after HCL diagnosis, which is the longest documented period between HCL diagnosis and subsequent bony involvement [30]. Rosen et al. reported a 54-year-old man who presented with a L5 vertebral body lesion and a lumbar epidural lesion extending from L3 to S2 [31]. Biopsy sampling of the epidural mass demonstrated CD20-positive B cells that expressed TRAP and CD25, consistent with a diagnosis of HCL. Bone marrow biopsy of the same patient demonstrated focal and interstitial involvement by HCL. After treatment with a 5-day course of cladribine, the patient's symptoms resolved. After a follow-up of 8 months, the patient was clinically stable with normal hematological parameters and no neurological symptoms. Spedini et al. described a 71-year-old man presenting with a lytic lesion in the femoral neck. His bone marrow biopsy showed nodular pattern of involvement with intracellular TRAP positivity suggestive of HCL. The patient was successfully treated with radiotherapy and interferon-*α* [32]. Contrary to the aforementioned reports in line with our observations, Lal et al. reported a rare HCL patient presenting with isolated skeletal disease occupying the left femur neck, left proximal femur, and both greater trochanters yet without bone marrow involvement or splenomegaly [10]. After a seven-day infusion of 2-chlorodeoxyadenosine, the femoral lesions showed marked improvement [10]. Karmali et al. described an unusual case presenting with isolated left hip involvement and no bone marrow infiltration, who was treated successfully with a 5-day course of cladribine [11]. Filippi et al. reviewed the role of radiation therapy for skeletal localizations of HCL. Skeletal lesions in HCL, with an estimated incidence of 3%, are mainly osteolytic, can occur at various sites, and are almost always symptomatic. Localized radiation therapy has been extensively used as effective palliative treatment in such cases, with different total doses and fractionation schedules [33]. Demanes et al. reported that radiation therapy in moderate doses (approximately 5000 rad in five weeks) is useful in patients with symptoms or with bone lesions at critical locations [29]. Quesada et al. reported that radiotherapy or chemotherapy provides palliation and may prevent further morbidity from bone lesions [4].

Treatment indications in HCL are cytopenias and infectious complications, bleeding, or painful splenomegaly. The mainstay of HCL treatment is two nucleoside analogues: pentostatin and cladribine [34–39]. These agents induce complete remission (CR) in more than 80% of patients with a median duration of progression-free survival (PFS) of over 10 years [40–43]. If the remission lasts more than two years, it is recommended to retreat with the same agent. If the remission duration is short, the use of an alternative agent is recommended. Combination of either of the nucleoside analogues with rituximab is suggested for those relapsing before two years [43, 44]. Also, rituximab 375 mg/m^2^ given weekly for 8 weeks is the best choice for patients refractory to treatment with nucleoside analogues [45]. Splenectomy can be considered if patients have symptomatic splenomegaly (>10 cm below costal margin) in the presence of minimal bone marrow involvement [3]. Choice of agent at relapse depends on duration of first remission. Asymptomatic patients or those with minimal cytopenias may not require immediate therapy at relapse.

The current case represents an unusual presentation of severe bone pain and multiple metastatic lesions. The B cells in the bone biopsy specimen were positive for CD20, LCA, CD79 alpha, TRAP, CD11c, CD68, and annexin. Also, a molecular marker, BRAF V600E mutation, was detected in our case and distinguished the disease from other B-cell malignancies. Bilateral posterior iliac crest bone marrow biopsies showed no disease infiltration. Also, no organomegaly was present on physical examination and MRI imaging. The final diagnosis was HCL with isolated skeletal involvement. To the best of our knowledge, there are limited HCL reports in the literature with isolated skeletal involvement [10, 11]. Therefore, there is no standard established treatment for this group. Our patient first underwent radiotherapy to both proximal femurs as palliative treatment as suggested before [29, 33]. Four weeks after completion of radiotherapy, we also administered a 7-day course of 0.1 mg/kg/day cladribine because of the presence of multiple metastatic lesions as in the case of isolated skeletal involvement by Lal et al. [10]. Our patient is still in continued clinical or radiographic remission 14 months after completion of chemotherapy. To conclude, skeletal involvement by HCL without coexisting bone marrow involvement should be included in the differential diagnosis of lytic-sclerotic bone lesions.

## Figures and Tables

**Table 1:** Clinical features of reported cases of HCL with skeletal involvement.

	Ref.	No. of cases	Type of lesions	Location	Bone marrow infiltration	Treatment
1	Quesada et al. [4]	Four	Osteolytic lesions, severe osteoporosis, and aseptic necrosis	Femoral head	Yes	RT (*n* = 3), daunorubicin (*n* = 1)

2	Demanes et al. [29]	Two	Osteolytic lesions	Right femoral neck, upper and lower thoracic vertebrae, L2 vertebral body	Yes	RT

3	Lembersky et al. [8]	Eight	Osteolytic lesions, multiple osteoporotic vertebral compression fractures	Axial skeleton, primarily the proximal femur	Yes	RT, interferon-*α*

4	Herold et al. [9]	Two	Osteolytic lesions, pathologic fracture, compression fracture, osteoblastic lesions	Right femoral head and neck, multiple thoracic and lumbar vertebral bodies, 12th thoracic vertebra	Yes	RT

5	Snell et al. [30]	One	Pathological fracture	Femur	Yes	Surgery

6	Rosen et al. [31]	One	Osseous lesions (not osteolytic)	L-5 vertebral body, lumbar epidural lesion extending from L-3 to S-2	Yes	Cladribine

7	Spedini et al. [32]	One	Osteolytic lesion	Femoral neck	Yes	RT and interferon-*α*

8	Lal et al. [10]	One	Marrow-based lesions (not osteolytic)	Left femur neck, left proximal femur, and both greater trochanters	No	Cladribine

9	Karmali et al. [11]	One	Localized skeletal disease (no fracture)	Left hip involving the inferior half of the femoral head and neck	No	Cladribine

11	Present case	One	Osteolytic and osteosclerotic lesions	Axial skeleton, sacrum, pelvis, and both femurs	No	RT and cladribine

Ref.: references, No.: number, and RT: radiation therapy.
